# Nicotinamide Mononucleotide Prevents Retinal Dysfunction in a Mouse Model of Retinal Ischemia/Reperfusion Injury

**DOI:** 10.3390/ijms231911228

**Published:** 2022-09-23

**Authors:** Deokho Lee, Yohei Tomita, Yukihiro Miwa, Ari Shinojima, Norimitsu Ban, Shintaro Yamaguchi, Ken Nishioka, Kazuno Negishi, Jun Yoshino, Toshihide Kurihara

**Affiliations:** 1 Laboratory of Photobiology, Keio University School of Medicine, Shinjuku-ku, Tokyo 160-8582, Japan; 2 Department of Ophthalmology, Keio University School of Medicine, Shinjuku-ku, Tokyo 160-8582, Japan; 3 Aichi Animal Eye Clinic, Nagoya 466-0827, Japan; 4 Department of Internal Medicine, Keio University School of Medicine, Shinjuku-ku, Tokyo 160-8582, Japan

**Keywords:** nicotinamide mononucleotide, retinal ischemia/reperfusion, oxidative stress, neuroprotection, inflammation

## Abstract

Retinal ischemia/reperfusion (I/R) injury can cause severe vision impairment. Retinal I/R injury is associated with pathological increases in reactive oxygen species and inflammation, resulting in retinal neuronal cell death. To date, effective therapies have not been developed. Nicotinamide mononucleotide (NMN), a key nicotinamide adenine dinucleotide (NAD^+^) intermediate, has been shown to exert neuroprotection for retinal diseases. However, it remains unclear whether NMN can prevent retinal I/R injury. Thus, we aimed to determine whether NMN therapy is useful for retinal I/R injury-induced retinal degeneration. One day after NMN intraperitoneal (IP) injection, adult mice were subjected to retinal I/R injury. Then, the mice were injected with NMN once every day for three days. Electroretinography and immunohistochemistry were used to measure retinal functional alterations and retinal inflammation, respectively. The protective effect of NMN administration was further examined using a retinal cell line, 661W, under CoCl_2_-induced oxidative stress conditions. NMN IP injection significantly suppressed retinal functional damage, as well as inflammation. NMN treatment showed protective effects against oxidative stress-induced cell death. The antioxidant pathway (*Nrf2* and *Hmox-1*) was activated by NMN treatment. In conclusion, NMN could be a promising preventive neuroprotective drug for ischemic retinopathy.

## 1. Introduction

Retinal ischemia/reperfusion (I/R) injury is involved in various retinal ischemic diseases, including diabetic retinopathy, glaucoma, and vascular ischemic retinopathy. Retinal I/R injury can cause pathological events, such as the induction of reactive oxygen species and retinal inflammation, ultimately leading to retinal neuronal cell death [1,2,3]. As effective therapeutics have not yet been found or developed for retinal I/R injury, research on searching for promising neuroprotective drugs to prevent and/or suppress retinal I/R injury has been attempted at the preclinical stage [4,5].

Nicotinamide mononucleotide (NMN) is one of the major intermediates of nicotinamide adenine dinucleotide (NAD^+^), a crucial co-enzyme for various cellular redox metabolisms, including cellular proliferation, DNA repair, and cell death/survival [6,7]. In this aspect, boosting NAD^+^ biosynthesis has been nominated as beneficial for age-related metabolic disorders and diseases, including obesity, insulin resistance, and diabetes [8]. When it comes to the eye, recent studies have demonstrated that NMN administration could protect against photoreceptor cell damage in rodent models of light-induced retinopathy or retinal detachment [9,10]. However, little is known about the preventive role of NMN for retinal I/R injury in mice.

Thus, in the present study, we aimed to determine whether NMN treatment could exert retinal protection in a mouse model of retinal I/R injury induced by acute transient elevation of intraocular pressure. Furthermore, we attempted to investigate how NMN treatment could show therapeutic effects using a retinal 661W in vitro system.

## 2. Results

### 2.1. NMN Treatment Prevents Retinal Dysfunction in a Mouse Model of Retinal I/R Injury Induced by Acute Elevation of Intraocular Pressure

**Figure 1:**
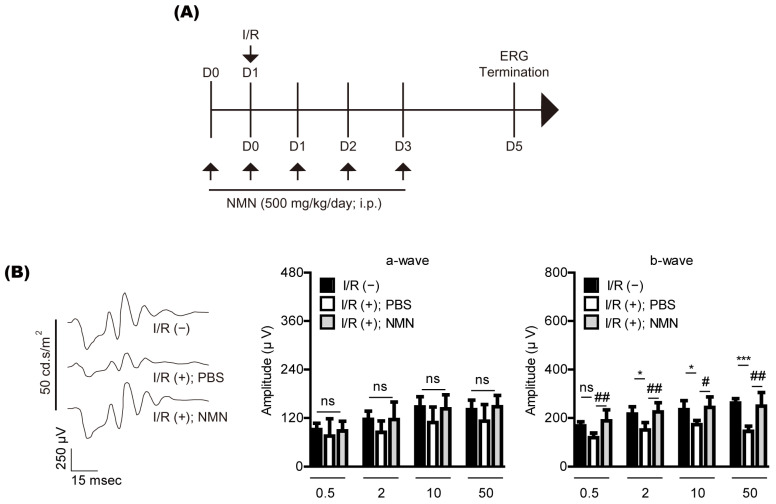
Retinal functional changes by nicotinamide mononucleotide (NMN) treatment in mice. (**A**) The schematic illustration shows whole experimental plans for NMN injection, retinal ischemia/reperfusion (I/R) injury, and the termination day of the whole experiment. i.p., intraperitoneal; D, day; ERG, electroretinography. (**B**) Representative waveforms (50 cd·s/m^2^) of ERG (*n* = 5–6 per group) demonstrated that the ERG amplitudes decreased 5 days after retinal I/R injury. NMN injection suppressed reductions in the ERG amplitude (especially b-wave), flashed with various intensities (0.5, 2, 10, or 50 cd·s/m^2^). * *p* <  0.05, *** *p* <  0.001, # *p* <  0.05, and ## *p* <  0.01. ns, not significant. The data were analyzed using one-way ANOVA followed by a Bonferroni post-hoc test. The data are drawn as the mean  ±  standard deviation.

To examine whether NMN treatment could prevent retinal dysfunction in a mouse model of retinal I/R injury, NMN was intraperitoneally injected into the mice one day before retinal I/R injury (Figure 1A). After injury, NMN was continuously intraperitoneally injected into the mice every day according to our experimental scheme. The dose of NMN was determined based on previous studies that evaluated various effects of NMN treatment [9,10,11,12]. We found that the amplitude of b-wave significantly decreased five days after retinal I/R injury, and its reduction was lessened by NMN treatment (Figure 1B).

### 2.2. NMN Treatment Reduces Retinal Inflammation in a Mouse Model of Retinal I/R Injury Induced by Acute Elevation of Intraocular Pressure

**Figure 2:**
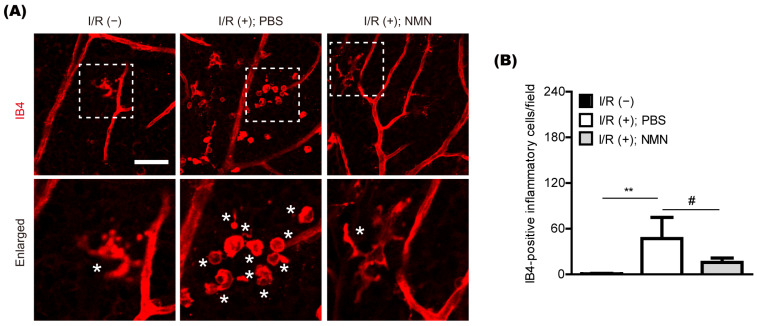
General modulation of retinal inflammation by nicotinamide mononucleotide (NMN) treatment in mice. (**A**,**B**) Representative pictures and quantitative analyses (*n* = 5–6 per group) demonstrated that the number of retinal I/R injury-induced IB4 positive inflammatory cells in the retina was reduced by NMN injection 5 days after injury. Scale bar: 50 μm. White boxes, enlarged pictures; Stars (*) in enlarged pictures, IB4 positive inflammatory cells. ** *p* <  0.01 and # *p* <  0.05. One-way ANOVA followed by a Bonferroni post-hoc test was used for the data analysis. Bar graphs are shown as the mean  ±  standard deviation. IB4, isolectin GS-IB4 from *Griffonia simplicifolia*.

Previously, we found pathologic increases in isolectin GS-IB4 (IB4)-positive inflammatory cells in the retina five days after retinal I/R injury [13]. Therefore, we examined whether the likelihood of its occurrence could be lessened by NMN treatment (Figure 2A,B). In our current system, the number of IB4-positive inflammatory cells in the ischemic retina was markedly reduced by NMN treatment.

### 2.3. NMN Treatment Exerts Neuroprotection in Retinal 661W Cells under CoCl_2_-Induced Oxidative Stress Conditions

**Figure 3:**
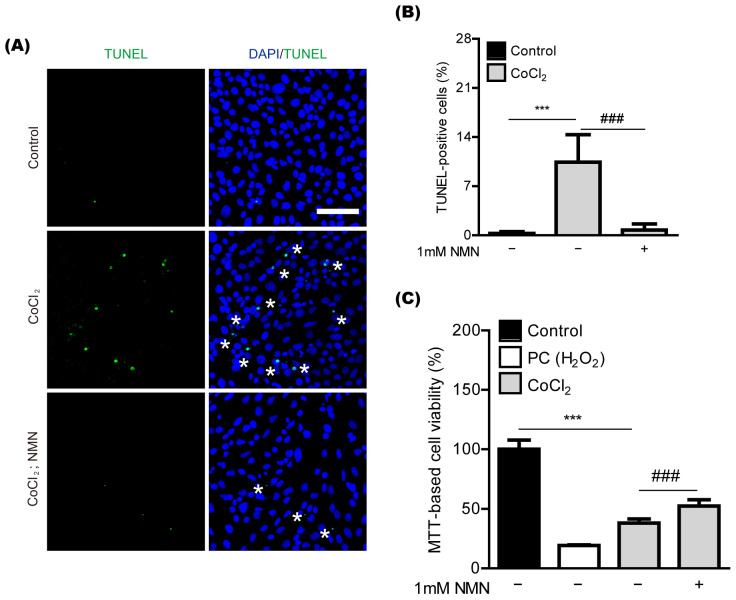
Neuroprotection in vitro by nicotinamide mononucleotide (NMN) treatment. (**A**,**B**) Representative pictures and quantitative analyses (*n* = 5 per group) demonstrated that the amount of oxidative stress-induced 661W cell death stained by TUNEL assay was reduced by 1 mM NMN treatment after 24 h of 400 μM of CoCl_2_ incubation. Scale bar: 100 μm. DAPI (blue); TUNEL (green); Stars (*) in pictures, TUNEL positive cells. *** *p* <  0.001 and ### *p* <  0.001. One-way ANOVA followed by a Bonferroni post-hoc test was used for the data analysis. Graphs are depicted as the mean  ±  standard deviation. (**C**) Quantitative analyses (*n* = 9 per group) demonstrated that MTT-based 661W cell viability increased by 1 mM NMN treatment after 10 h of 400 μM of CoCl_2_ incubation. *** *p* <  0.001 and ### *p* <  0.001. One-way ANOVA followed by a Bonferroni post-hoc test was used for the data analysis. Bar graphs are shown as the mean  ±  standard deviation. PC, positive control (250 μM of H_2_O_2_).

To further determine whether NMN treatment could show protective effects in retinal neuronal cells, we applied an in vitro system to our current study (Figure 3). 661W cells, a mouse-derived photoreceptor cell line possessing features of retinal ganglion precursor-like cells, were used, as they are generally used as one of the in vitro models for studying retinal degeneration [14,15,16]. Previously, we found that CoCl_2_-induced oxidative stress could cause 661W cell death [17]. In our current system, we also found 661W cell death by CoCl_2_-induced oxidative stress, analyzed with TUNEL assay (Figure 3A,B). NMN treatment significantly reduced oxidative stress-induced 661W cell death. This outcome was further confirmed with MTT assay (Figure 3C).

### 2.4. NMN Treatment Upregulates the Antioxidant Genes in Retinal 661W Cells under CoCl_2_-Induced Oxidative Stress Conditions

**Figure 4:**
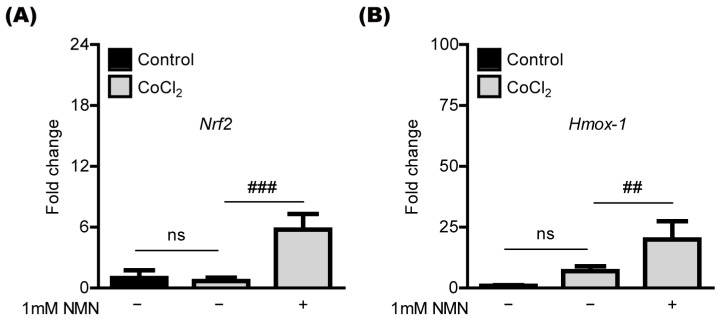
Antioxidant gene regulation by nicotinamide mononucleotide (NMN) treatment. (**A**,**B**) Quantitative analyses (*n* = 5 per group) demonstrated that NMN treatment (6 h) increases mRNA expression in the antioxidant genes (*Nrf2* and *Hmox-1*) in 661W cells under 400 μM CoCl_2_-induced oxidative stress conditions. ## *p* <  0.01 and ### *p* <  0.001. ns, not significant. The data were analyzed using one-way ANOVA followed by a Bonferroni post-hoc test and drawn as the mean  ±  standard deviation.

NMN treatment has been shown to increase antioxidant genes such as *Nrf2* and *Hmox-1* in various cell types [9,10,11,18,19]. Thus, we applied its concept to our current system (Figure 4). Under the same conditions of CoCl_2_-induced oxidative stress above, we found that the expression of *Nrf2* and *Hmox-1* mRNA was markedly increased by NMN treatment in 661W cells (Figure 4A,B).

## 3. Discussion

The current study demonstrated that retinal neuronal cells could be damaged by retinal I/R injury, and consecutive NMN treatment could show preventive effects against such injury. The therapeutic effects of NMN were further examined using the 661W cell line. Oxidative stress-induced 661W cell death was markedly decreased by NMN treatment, with increases in antioxidant gene expression (*Nrf2* and *Hmox-1*). Although NMN therapy has gradually been reported to be beneficial for eye diseases [20,21], as far as we know, this report is the first to expand the role of NMN in a mouse model of retinal I/R injury induced by acute increases in intraocular pressure.

NMN is a key intermediate of NAD^+^, an important metabolic redox co-enzyme in most eukaryotic cells. NMN supplements could be involved in various biological processes such as aging, cell growth, cell death and protection, and DNA repair [22,23]. To date, the therapeutic outcomes of treating NMN or targeting its related NAD^+^ pathways have been unraveled in various experimental models of metabolic diseases and disorders, including diabetes, obesity, ischemia/reperfusion injury, heart failure, vascular dysfunction, hemorrhage, cognitive dysfunction, kidney injury, and alcoholic liver disease [22,23,24,25,26]. This indicates that NMN and its related NAD^+^ pathways might be crucial for regulating multiple cellular functions in the body.

Based on our current data, retinal I/R-induced retinal dysfunction and oxidative stress-induced cell death were reduced by NMN treatment. Other groups have reported similar effects. Chen et al. demonstrated that NMN supplementation reduces photoreceptor cell loss in the early phase of retinal detachment [10]. They further found that NMN treatment exerts neuroprotection in 661W cells under tBuOOH-induced oxidative stress conditions (tBuOOH; one of the most widely used inducers of oxidative stress [27,28,29]). Lin et al. showed that NMN treatment protects against retinal dysfunction in mice lacking nicotinamide phosphoribosyltransferase (*Nampt*; a rate-limiting enzyme in the NAD^+^ salvage biosynthesis pathway [30,31]) and mice with light-induced retinopathy [9]. They further found that NAMPT inhibitor FK866-induced cell death was decreased by NMN treatment in 661W cells. In this regard, our current results are consistent with those of other previous reports, which implies that NMN could have therapeutic effects against retinal cell damage.

Previous reports from us and others have suggested that reductions in pathologic retinal inflammatory cells could be beneficial for retinal neuronal protection in a murine model of retinal I/R injury [13,32,33]. The therapeutic strategy of modulating pathologic inflammatory cells for neuroprotection can be seen in brain injury, rather than just retinal I/R injury [34,35,36]. Our current system detected reductions in pathologic retinal inflammatory cells in the NMN-treated retinas. Chen et al. also found that NMN supplementation could suppress retinal inflammation in retinal detachment [10]. Wei et al. demonstrated that NMN treatment could suppress neuroinflammation to exert neuroprotection in intracerebral hemorrhage [18]. Although the way in which NMN treatment is involved in modulating inflammation requires further investigation, its effect might contribute to retinal protection against retinal I/R injury.

NMN is widely known to be associated with antioxidants to protect cells against free radicals [37]. Based on previous reports, the antioxidant function of NMN has been linked to the *Nrf2*/*Hmox-1;Ho-1* antioxidant signaling pathway [9,10,11,18,19,38]. Pu et al. demonstrated that NMN treatment could increase cell viability and restore tight junctions in human corneal epithelial cells through the *Nrf2*/*Hmox-1* pathway [19]. Luo et al. showed that NMN administration could restore redox homeostasis in a murine model of oxidative stress-induced liver injury through the *Nrf2* pathway [39]. Wei et al. showed that NMN treatment could attenuate brain damage induced by intracerebral hemorrhage through the *Nrf2*/*Hmox-1* pathway [18]. Chen et al. demonstrated that NMN treatment could increase HO-1 protein expression in 661W cells under tBuOOH-induced oxidative stress conditions [10]. Taken together, our current data also support the notion that the therapeutic role of NMN could be involved in the *Nrf2*/*Hmox-1* antioxidant signaling pathway in various eukaryotic cells.

In summary, we applied the promising NMN therapy to retinal I/R injury in the current study. We further found that NMN treatment could protect against oxidative stress-induced retinal cell death and could upregulate the antioxidant pathway (*Nrf2* and *Hmox-1*) in retinal cells. Although more studies (such as unraveling the clear mode of action of NMN treatment in ischemic eyes, including the retina, choroid, and retinal pigment epithelium) are needed, we suggest a promising NMN therapy for ischemic retinopathy based on our short current summary.

## 4. Materials and Methods

### 4.1. Animal and Retinal Ischemia/Reperfusion (I/R) Injury

The mouse experimental processes of the Ethics Committee on Animal Research of Keio University School of Medicine (#16017), the ARVO Statement for the Use of Animals in Ophthalmic and Vision Research, and the International Standards of Animal Care and Use, Animal Research: Reporting in Vivo Experiments were followed. Adult male mice (C57BL/6, 6–8 weeks old) were bought from CLEA Japan (Tokyo, Japan). After mouse randomization, retinal I/R injury was induced in their eyes, as described in our previous paper [13]. Briefly, anterior chamber cannulation was performed using a 35-gauge needle, and high intraocular pressure was maintained by PBS solution for 40 min. After injury, the mice were recovered with hot pads to maintain their body temperature and then moved back to their cages for further experiments.

### 4.2. Electroretinography (ERG)

General scotopic ERG was performed as described in our previous retinal I/R injury study [13]. After 24 h of dark adaptation, the mice were placed on a Ganzfeld dome table under dimly lit conditions. After pupil dilation followed by anesthesia (midazolam, medetomidine, and butorphanol tartrate, called MMB [40]), active ERG electrodes, connected to a PuREC acquisition system (MAYO, Inazawa, Japan), were softly contacted with the mouse cornea. Then, the general scotopic ERG amplitudes (a- and b-waves) were obtained at standard flash intensities.

### 4.3. Immunohistochemistry (IHC)

IHC was performed as described in our previous retinal I/R injury study [13]. Briefly, after paraformaldehyde (4% PFA) fixation for more than 3 h, the mouse eyeballs were moved to a Petri dish with cold PBS. After the retinas were collected from the eyeballs, the retinal samples were flat-mounted using micro-scissors and incubated in IB4 solution for 24 h. After washing with cold PBS several times, the retinal samples were mounted and examined by an LSM710 fluorescent microscope (Carl Zeiss, Jena, Germany).

### 4.4. Cell Culture

Retinal 661W cells were generally cultured in DMEM (Cat #08456-36, Nacalai Tesque, Kyoto, Japan) media with 10% FBS and 1% streptomycin–penicillin as described in our previous papers [41,42]. The cell culture was maintained under atmospheric conditions containing 5% CO_2_ at 37 °C.

### 4.5. Terminal Deoxynucleotidyl Transferase dUTP Nick end Labeling (TUNEL) and MTT Assays

A TUNEL assay was conducted as described in our previous papers [13,43]. We basically followed the manufacturer’s manual instructions (in situ Apoptosis Detection Kit, Cat #MK500, Takara Bio, Japan). PFA (4%)-fixed 661W cells were subjected to permeabilization using a permeabilization buffer supplied from the assay kit for 5 min on ice. A labeling reaction mixture with TdT enzymes was added to the cells for 1 h. Then, the DAPI solution was incubated for 1 min to stain the nuclei. The samples were mounted and investigated using an LSM710 fluorescent microscope (Carl Zeiss, Jena, Germany).

An MTT assay was also performed as described in our previous report [41]. The MTT solution (Cat #M2128, Sigma, St. Louis, MO, USA) was given to each well for 2 h. After removing the supernatant, DMSO was added to each well. Then, color absorbance was measured and determined using a microplate reader (Synergy HT Multi-Mode, Winooski, VT, USA).

### 4.6. Quantitative PCR (qPCR)

All steps for quantitative PCR (qPCR) were described in our previous reports [42,44]. Briefly, RNA extraction, cDNA synthesis, and qPCR were performed with each kit (Qiagen, Velno, Netherlands; TOYOBO, Osaka, Japan; Applied Biosystems, Waltham, MA, USA, respectively). The primer information used in our current study is outlined in Table 1. The general ΔΔ*CT* calculation method was applied for our qPCR analysis.

### 4.7. Statistical Analysis

All values in our current data were depicted as the mean ± standard deviation. Statistical significance was determined using one-way ANOVA followed by a Bonferroni post-hoc test. Statistical significance was considered when the *p*-value < 0.05.

## Figures and Tables

**Table 1:** Primer list.

Name	Direction	Sequence (5′→3′)	Accession Number
*Hprt*	Forward	TCAGTCAACGGGGGACATAAA	NM_013556.2
	Reverse	GGGGCTGTACTGCTTAACCAG	
*Hmox-1*	Forward	CACTCTGGAGATGACACCTGAG	NM_010442.2
	Reverse	GTGTTCCTCTGTCAGCATCACC	
*Nrf2*	Forward	TAGATGACCATGAGTCGCTTGC	NM_010902.4
	Reverse	GCCAAACTTGCTCCATGTCC	

## Data Availability

The current study’s data are available upon request from the corresponding author.
